# Phosphoglycerate Mutases Function as Reverse Regulated Isoenzymes in *Synechococcus elongatus* PCC 7942

**DOI:** 10.1371/journal.pone.0058281

**Published:** 2013-03-06

**Authors:** Jiri Jablonsky, Martin Hagemann, Doreen Schwarz, Olaf Wolkenhauer

**Affiliations:** 1 Department of Systems Biology and Bioinformatics, University of Rostock, Rostock, Germany; 2 Department of Plant Physiology, University of Rostock, Rostock, Germany; Universidad de La Laguna, Spain

## Abstract

Phosphoglycerate-mutase (PGM) is an ubiquitous glycolytic enzyme, which in eukaryotic cells can be found in different compartments. In prokaryotic cells, several PGMs are annotated/localized in one compartment. The identification and functional characterization of PGMs in prokaryotes is therefore important for better understanding of metabolic regulation. Here we introduce a method, based on a multi-level kinetic model of the primary carbon metabolism in cyanobacterium *Synechococcus elongatus* PCC 7942, that allows the identification of a specific function for a particular PGM. The strategy employs multiple parameter estimation runs in high CO_2_, combined with simulations testing a broad range of kinetic parameters against the changes in transcript levels of annotated PGMs. Simulations are evaluated for a match in metabolic level in low CO_2_, to reveal trends that can be linked to the function of a particular PGM. A one-isoenzyme scenario shows that PGM2 is a major regulator of glycolysis, while PGM1 and PGM4 make the system robust against environmental changes. Strikingly, combining two PGMs with reverse transcriptional regulation allows both features. A conclusion arising from our analysis is that a two-enzyme PGM system is required to regulate the flux between glycolysis and the Calvin-Benson cycle, while an additional PGM increases the robustness of the system.

## Introduction

Phosphoglycerate-mutases (PGMs) are a group of non-homologous glycolytic enzymes [1], having independent evolutionary origins, catalyzing the reversible conversion of 3-phosphoglycerate (3PGA) to 2-phosphoglycerate (2PGA). PGMs can be found in different compartments in the eukaryotic cells fulfilling different tasks [2]. PGMs are also present in prokaryotes [3] such as cyanobacteria, in which the different PGM isoenzymes are localized in one compartment. PGMs can be divided into two analogous subgroups, cofactor-independent and cofactor (2,3-bisphosphoglycerate)-dependent enzymes [3]. The occurrence of these enzyme types seems to be scattered among prokaryotes. Based on our own analysis, the presence of PGM proteins from these two groups is unpredictable among cyanobacteria, as has been reported before for other bacteria [1]. This scattered occurrence seems to imply that the type of PGM has no significant role in the regulation of the system. However, the occurrence of more than one PGM in one compartment certainly needs some regulation or functional specialization, unknown so far.

**Figure 1:**
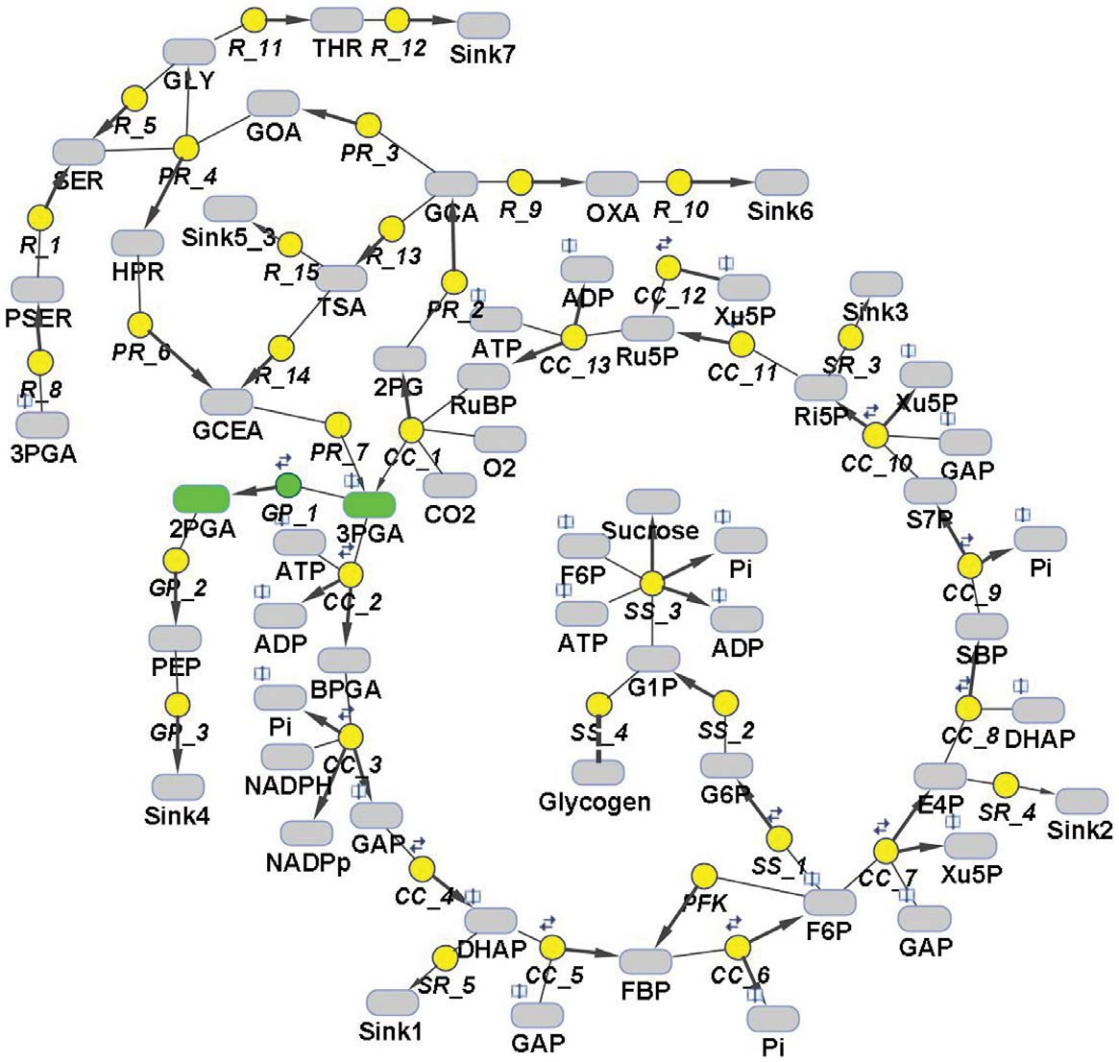
Scheme of the primary carbon metabolism, encoded as a kinetic model of *Synechococcus elongatus* PCC 7942. The model includes the Calvin-Benson cycle, sucrose and glycogen synthesis, photorespiratory pathways, glycolysis and sink reactions, representing the adjacent pathways. Green color represents the reaction catalyzed by phosphoglycerate mutase (PGM) and indicates its cardinal position in the crossroads of metabolic pathways (need for complex model). Note: The reactions are described in the model by reversible and irreversible Michaelis-Menten kinetics; reversibility of particular reaction is indicated by two little arrows.

The preferred substrate of PGM is 3-phosphoglycerate (3PGA). In cyanobacteria, 3PGA is made by photosynthetic CO_2_ fixation, in which 3PGA represents the first stable carbon-fixation product of the Calvin-Benson cycle. The majority of 3PGA is used for the regeneration of the Calvin-Benson cycle, CO_2_ acceptor molecule ribulose-bisphophate. Excess carbon is taken out of the cycle and stored as glycogen or shuttled into the primary carbon metabolism via glycolysis (Fig. 1). The flux of 3PGA in the compartmented cell of land plants is regulated by phosphate translocator (chloroplast ↔ cytosol), which is not present in cyanobacteria. In the non-compartmented cyanobacterial cell, PGMs could be the key regulator of the flux out of the Calvin-Benson cycle. Marked changes in the relative flux of carbon through the Calvin-Benson cycle and its export into the glycolytic path have been observed in cyanobacteria exposed to excess or limited amounts of inorganic carbon [4], [5].

**Figure 2:**
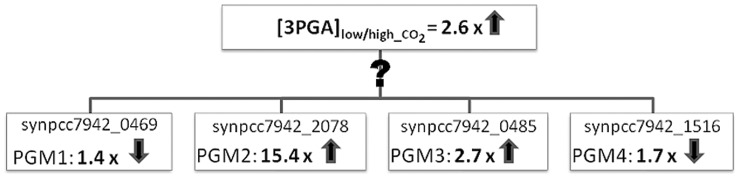
Comparison of fold changes in concentration for 3PGA and in expression levels of the four annotated PGM isoenzymes in cells of *Synechococcus elongatus* PCC 7942 after shifting from high to low CO_2_ level. Note: synpcc7942_0485 probably represents a gene encoding a phosphoserine phosphatase but we cannot exclude if it functions as PGM.

Steady state metabolomic and transcriptomic analysis with the model cyanobacterium *Synechococcus elonagtus* PCC 7942 (hereafter *Synechococcus*) indicated that cells shifted to low CO_2_ availability reduce Calvin-Benson cycle activity, stop glycogen storage, and increase the export of organic carbon through the glycolysis [6]. In the *Synechococcus* genome (http://genome.kazusa.or.jp/cyanobase/SYNPCC7942) four PGM isoenzymes are annotated (Fig. 2). These annotations are mainly based on sequence similarities determined with the BLAST algorithm [7]. However, automated BLAST-based annotation can provide false functional assignments [8]. In the case of *Synechococcus,* the occurrence of isoenzymes in one compartment suggests a regulatory/specialized role of these isoenzymes in central metabolism. Moreover, in the cyanobacterial carbon metabolism, PGMs have a cardinal position at the crossroads of Calvin-Benson cycle and associated carbon metabolism via glycolysis (Fig. 1). Therefore, it is important to understand the regulation of PGMs and thereby to validate their annotation.

We here propose the use of kinetic modeling for such analysis. Kinetic modeling is a standard method for predicting the behavior of biological systems [9]. However, reactions catalyzed by isoenzymes are commonly described by single enzymatic kinetics, which would not explain hidden regulatory mechanisms. In order to address this challenge, we have designed a multi-level kinetic model of carbon core metabolism of *Synechococcus*. The model not only helps in the validation of the annotation but also in the explanation of regulatory mechanism for PGM isoforms in one compartment.

## Materials and Methods

The model was developed and simulations were executed using the SimBiology toolbox of MATLAB (Mathworks Inc.). The routine employed for parameter estimation was a hybrid genetic algorithm (ga_hybrid, Mathworks Inc.). The model is available in the supplement (Model S1).

### Systems Biology Workflow

The kinetic model, a successor of the corrected Zhu model [10], [11] of the Calvin-Benson cycle, was redesigned for cyanobacteria, extended (photorespiratory pathways, glycolysis) and validated on available metabolic data from *Synechococcus el.* PCC 7942 [6]. The constraints of the model are: 1) The ATP · (ADP +ATP)^−1^ ratio was maintained in the physiological range 0.74–0.76 [12], both in high and low CO_2_ steady states and 2) the biomass production in high CO_2_ was calculated from growth rate data. On average, *Synechococcus* shows a 3.4-fold higher growth at 5% CO_2_ (defined as high CO_2_, [6]) compared to ambient air CO_2_ (defined as low CO_2_, [6]).

The parameter estimation for up to four annotated isoenzymes of PGM was run in high CO_2_ steady state and the result (kinetic parameters) stored if the difference between the simulated and experimental metabolic data was lower than 15%. We also assumed that, in the case of isoenzymes in non-compartmented prokaryotic cell, a change in gene expression in the steady state is equal to changes in protein activity, i.e., minimal or no post-translation modifications. For the unidentified part of carbon metabolism - reaction catalyzed by PGM - four transcriptomic profiles (four PGM isoforms) were tested for combinations of one to four isoforms and the quality of match with metabolic data in low CO_2_ was stored for each profile.

### Experimental Data

Relative transcriptomic and metabolomics data of high CO_2_ as well as low CO_2_ *Synechococcus* cells were taken from [6]. A considering of two environmental conditions was necessary to known and implement the changes in transcriptomic level of PGMs in the model and helpful for constraining the model by doubling the amount of metabolic data. For 3PGA, cellular concentrations were calculated using the data from [13]. These authors reported that cyanobacteria contain 1300 nmol 3PGA/g fresh weight. According to our calibration of cyanobacterial fresh weight and total cellular volume, this amount corresponds to 5.39 mM 3PGA in the total cell volume in low CO_2_ grown cells. The concentration of 2-phosphoglycerate was recalculated on the basis of known ratio to 3PGA [6].

## Results and Discussion

### When One Enzyme is not Enough

The implementation of isoenzymes within a kinetic model, when localized in one compartment, deals with two extremes. First, for only one enzyme and the model not being constrained by a lack of data, there are still non-identifiability problems due to multiple sets of parameter value combinations that are equally able to fit the data related to a particular state. Second, considering two or more enzymes, the combinational explosion in the parameter space leads to computational requirements that make it virtually impossible to fit the model to sets of experimental data for different states of the system.

Various studies have focused on the response of cyanobacteria to changes in the environment, by considering a range of omics data [6], [14], [15], which allow constraining the model. On the basis of these results, we were aiming to develop a method that would permit the identification and analysis of isoenzymes in a one-compartment system and within a reasonable amount of time (days to weeks). We here particularly focus on PGMs because of their key position in regulating the flux of carbon through the Calvin-Benson cycle and its export into glycolysis in cyanobacteria. This position of PGMs in the metabolic network makes it necessary to establish a complex model (Fig. 1).

We started with the simplest scenario, encoding only one PGM, which is a common approach to un-constrained kinetic modeling. Although this approach cannot reveal anything about the regulation of catalyzed reactions in the un-constrained model, it is yet unknown if the outcome based on the constrained model can reveal some additional information about the system regulation or not. We therefore run a set of independent parameter estimations in high CO_2_ for a single PGM and used these values in numerical simulations. The quality of the estimates is evaluated by comparing the quality of match of simulations with metabolic data, separately for preferred substrate, 3-phosphoglycerate (3PGA) and product, 2-phosphoglycerate (2PGA), in low CO_2_ (for details see Materials and Methods).

**Figure 3:**
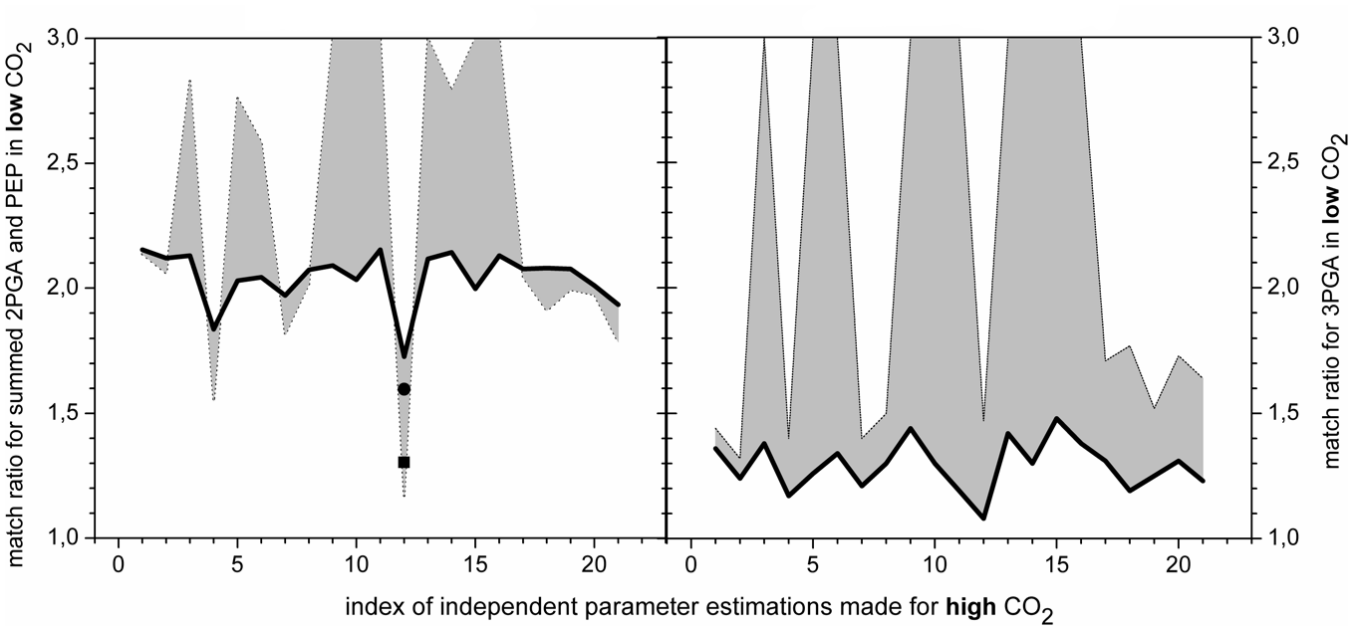
Quality for the match of simulated and measured data in low CO_2_ for single PGM scenario in dependence on (i) varying PGM activities fitted in high CO_2_ and (ii) regulation by transcript amounts. Figures represent the match between simulated and measured data in low CO_2_ in dependence of estimated kinetic parameters (V_max_ and k_M_ values for preferred substrate and product) in high CO_2_ for a single PGM scenario.The V_max_, fitted to steady state in high CO_2_, was modified by the amount of PGM isoforms taken from the changes in mRNA values (one by one) after shift from high to low CO_2_. Results are shown for randomly chosen set of twenty parameters estimation runs for PGM and the best fit (Nr. 12). The **left figure** shows the match for preferred product of PGM, 2-phosphoglycerate (2PGA). The black solid line shows the impact of 1.7-fold down-regulated enzyme, corresponding to PGM4; gray contours indicate the difference in matching the data if PGM is 15.4-fold up-regulated (corresponding to PGM2). In order to illustrate the impact of transcriptomic changes for the other two annotated PGMs, the results for 1.4-fold down-regulated (circle) and 2.7-fold up-regulated (square) isoenzymes are presented in the case of the best fit. The **right figure** shows the match for preferred substrate of PGM, 3-phosphoglycerate (3PGA); colors/lines have the same meaning as for the figure on the left. Notes: 1) the top boundary of axis y shows results equal or worse than match ratio equals to 3, 2) The match ratio is calculated as X/Y where X(Y) is a higher(lower) number from a pair of simulated and experimental values for a particular data point, 3) Fit from high CO_2_ was included (saved as a result) if the difference between simulated and measured data was smaller than 15%, 4) each point represents an independent simulation run compared to experimental data - the lines improve the perception for the differences in match ratios and have no other meaning.

**Figure 4:**
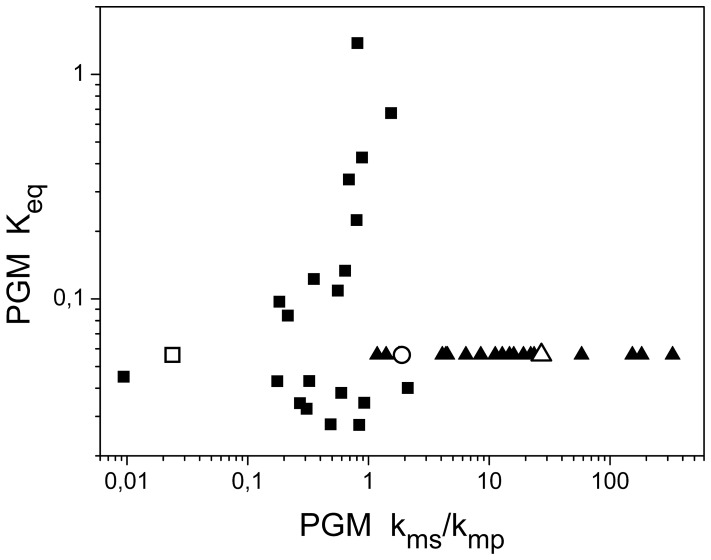
Parameter space of the equilibrium constant and ratio of k_M_ constants for single PGM and dual PGMs scenarios. The values k_ms_ and k_mp_ indicate the k_M_ values for the preferred substrate and product (reversibility), respectively. Keq indicates the equilibrium constant. **Solid squares** denote independent runs of parameter estimation, presented in Fig. 3, for single PGM scenario. **The open square** denotes the best fit for single PGM scenario. **Solid triangles** denote independent runs of parameter estimation, presented in Fig. 5, for dual reverse regulated PGMs scenario for PGM beta. **The open triangle** denotes the best fit for dual reverse regulated PGMs scenario for PGM beta. **The open circle** denotes the best fit for triple PGMs scenario for PGM gamma. This analysis shows how multiple sets of kinetic parameters match experimental data in one steady state for unconstrainted parameter estimation.

The results are striking. The analysis reveals two trends, showing that one PGM provides a match either for preferred substrate or preferred product (Fig. 3) but never for both. Moreover, these trends indicate an impact of transcriptomic regulation, which affects V_max_ in the enzymatic kinetics that describes the PGM. The first trend, exhibiting robustness in the system behavior (Fig. 3, black solid line), is associated with down-regulated PGM4. The other one (Fig. 3, gray contours), connected with up-regulated PGM2, shows a high sensitivity towards variations in the kinetic parameters (K_eq_ and k_M_). This pattern was tested for a broad range of K_eq_ and k_M_ values (Fig. 4, solid squares). Furthermore, we can observe both, a very good and bad match, with experimental data (Fig. 3, gray contours), implying a major role in the kinetic regulation of the system. Taken together, single PGM cannot regulate the interconversion between 3PGA and 2PGA, however, the single isoenzyme scenario has provided valuable information about the requirements for homeostasis of this metabolic crossroad.

### Reverse Regulated Versus Co-regulated Isoenzymes

We have shown that the one-isoenzyme scenario cannot keep the expected balance between 3PGA and 2PGA (Fig. 3). We can either get a robust system, which is however out of the physiological range, or a system that is very sensitive to changes in the parameter values. The natural next step in the analysis is to test for two PGMs in one compartment. This approach requires metabolic data from two steady states and transcriptomic data describing the shift between these two states, in our case, from high to low CO_2_. However, parameter estimation for two isoenzymes, running for two steady states of metabolism, has enormous computational demands. At this point, data collected from the single PGM scenario, based on the constrained model, proved to be very useful.

The single PGM scenario suggests that it is the up-regulated PGM2 which is likely to provide the kinetic regulation for the reaction due to high sensitivity with respect to kinetic parameter values. We therefore tested the dual PGM scenario with two PGMs, denoted as alpha and beta. We employed the parameter values from the best fit provided by the single PGM scenario for alpha PGM (Fig. 4, open square) and varied the parameters for beta PGM, see Fig. 4 (solid triangles); the equilibrium constant is the same for both PGMs. Moreover, for the sake of comparison, we have added the robust case from the single PGM scenario (1.7-fold down-regulated PGM4) and another dual PGM scenario in which both PGMs were co-down-regulated (PGM1 and PGM4).

**Figure 5:**
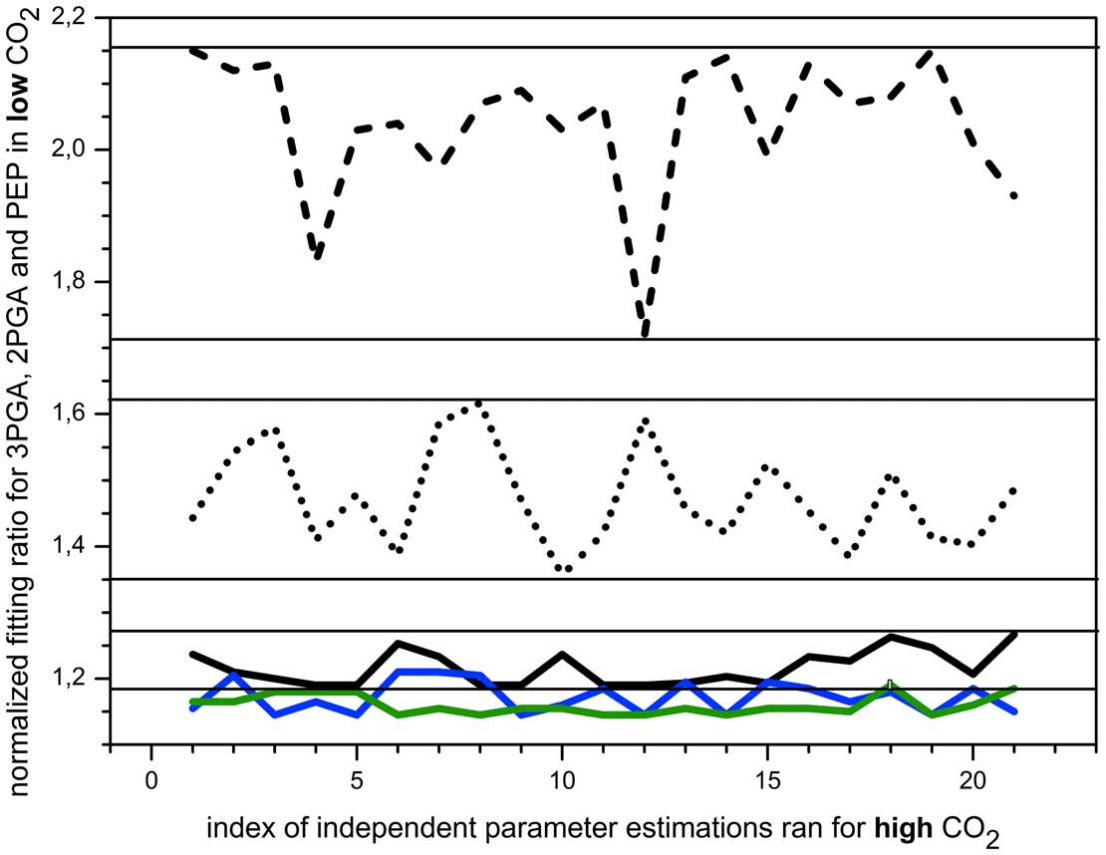
Impact of reverse and similar transcriptional regulation of PGM isoenzyme genes in one compartment. For the purpose of comparison, the dashed line represents the single enzyme scenario from Fig. 3, 1.7-fold down-regulated PGM4 (merged solid lines from Figure 3). Further, two dual PGMs scenarios are presented. K_eq_ and k_M_ values for alpha PGM are from the best fit for single PGM scenario (Fig. 4, open square) and V_max_ for alpha PGM and all kinetic parameters for PGM beta were estimated to fit the experimental data in high CO_2_ steady state. Alpha and beta stand for two PGMs in dual PGMs scenario. The dotted line shows the cooperation of co-regulated PGM4 and PGM1 (1.7- and 1.4-fold down-regulation, respectively). The solid line indicates the case in which reverse regulation of PGM2 and PGM4 takes a place (15.4-fold up-regulation and 1.7-fold down-regulation). The blue line indicates the scenario of three PGMs in which PGM1 (1.4-fold down-regulation) is considered as gamma PGM. The k_M_ values for beta PGM are taken from the best fit of dual reverse PGMs scenario (Fig. 4, open triangle). The green line indicates another triple PGMs scenario where k_M_ values, both for beta and gamma PGMs, were varied. Note: each point represents an independent simulation run compared to experimental data - the lines improve the perception for the differences in match ratios and have no other meaning.

The comparison with other scenarios clearly shows a large improvement, both in robustness against varying parameter values for the beta PGM and in the ability to describe the experimental data if we proceed from single PGM scenario (Fig. 5, dashed line), over dual co-regulated PGMs (Fig. 5, dotted line) to dual reverse regulated PGMs (Fig. 5, solid line). It is therefore the combination of two PGMs with reverse transcriptomic regulation, PGM2 and PGM4, giving the closest match with experimental data. Moreover, if we have a look at the estimated parameters for the best fit for this scenario (Fig. 4, open square and open triangle), the Michaelis constants for preferred substrate and product for alpha and beta PGMs in Table 1 have nearly interchanged values. This is an expected outcome from a evolutionary point of view for two enzymes catalyzing the same reaction in one compartment, thus supporting the applied approach. In summary, our in silico experiment led us to the conclusion that reverse gene regulation for two isoenzymes in one compartment, together with the opposite affinity for the substrate and product, provides very good explanation of the measured metabolic data.

**Table 1:** Estimated kinetic parameters for the best fit describing the reverse regulated dual PGMs scenario.

kinetic parameter	value	unit
Keq	0.056	dimensionless
**Vmax_alpha**	0.050	mM • s^−1^
kms_alpha	0.042	mM
kmp_alpha	1.772	mM
**Vmax_beta**	0.027	mM • s^−1^
kms_beta	1.257	mM
kmp_beta	0.047	mM

Activity of PGMs is in the model described by reversible Michaelis-Menten kinetics. Note: V_max_ values are normalized to the activity of RuBisCO. The routine employed for parameter estimation was a hybrid genetic algorithm.

The results clearly show high robustness of the system (Fig. 5, solid line) against varying the kinetic parameters (Fig. 4, solid triangles), which is equivalent to noise from fluctuating metabolite concentrations in a single cell. Hence the results support our initial assumption that system of isoenzymes does not require an additional regulatory mechanism, for instance the post-translational modifications. This was implicit in the assumed 1∶1 ratio between the transcript level and protein activity for isoenzymes in the non-compartmented cell.

### The Case of Two other Annotated PGMs

We have shown that two reverse regulated PGMs are able to explain the experimental data (Fig. 5), i.e., to regulate the interconversion between 3PGA and 2PGA and thus the flux between the Calvin-Benson cycle and glycolysis. This raises the questions whether there is any benefit of having more than two PGMs in one compartment. In order to test the triple PGMs scenario, we have taken the best fits for alpha and beta PGMs (Fig. 4, open square and open triangle) and varied the k_M_ values for gamma PGM. Our analysis of the triple PGMs scenario shows that there is a negligible improvement in robustness and only a small improvement in matching experimental data (Fig. 5, blue line). The k_M_ values for gamma PGM, in the case of the best fit, is in between the k_M_ values of PGMs alpha and beta (Fig. 4, open circle). There was however no improvement in the biomass production (our estimation of growth speed), which implies that only two PGMs are necessary to control the flux between the Calvin-Benson cycle and glycolysis in the controlled environment. Since two-component response regulators have been proposed for the glycolytic pathway [16], this further supports our conclusion that only two PGMs are essential for the regulation of the system.

**Figure 6:**
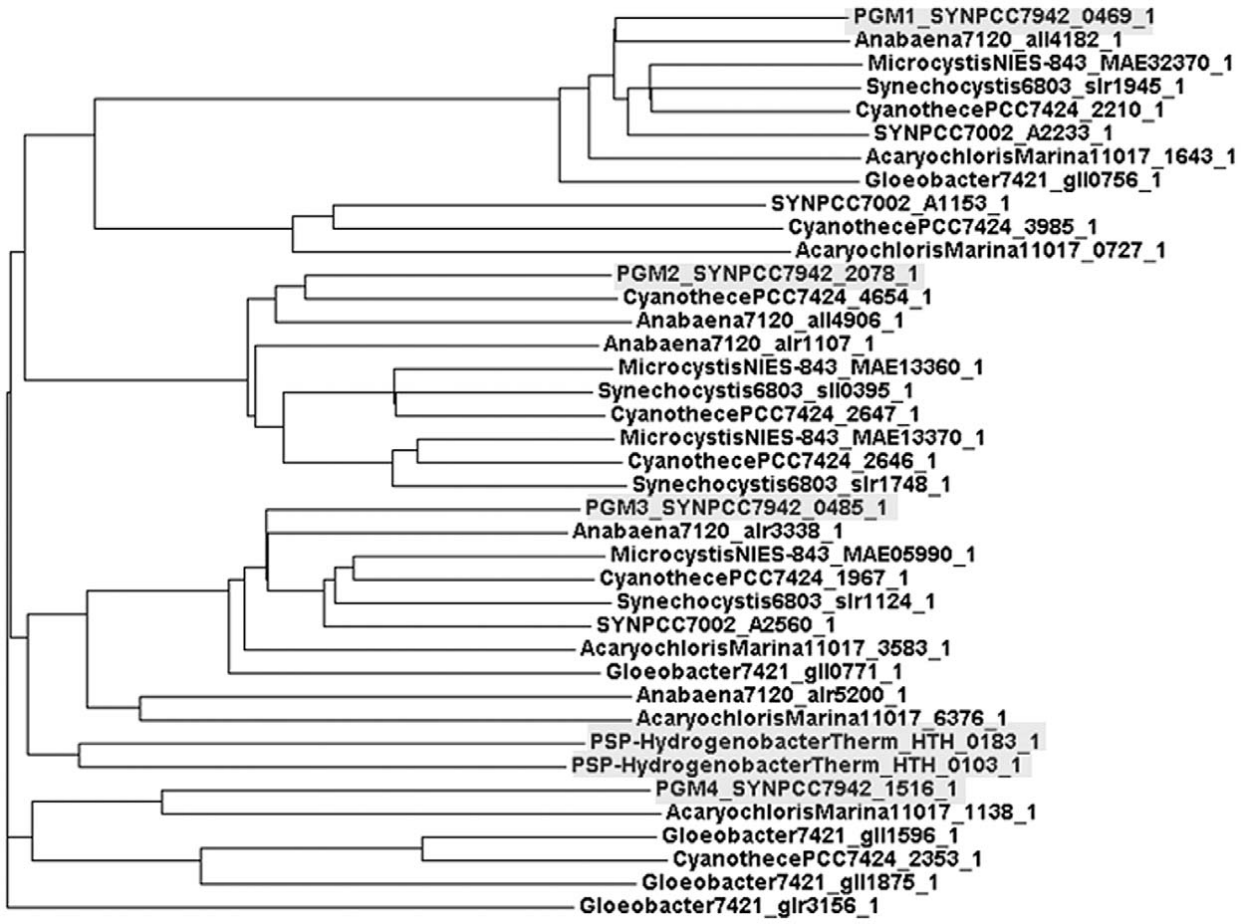
Grouping of PGMs and PSPs by using cluster analysis. ClustalW2 2.1 (http://www.ebi.ac.uk/Tools/) was employed as a tool for protein alignment analysis, codon table for bacteria was selected. PGMs 1–4 (*Synechococcus*) and two PSPs (*Hydrogenobacter thermophilus*) are highlighted. PGM3 is clustered with two PSPs.

Recently, two assumed members of PGM family, cofactor dependent PGMs annotated in *Hydrogenobacter thermophilus*, were identified in a wet lab experiment as a phosphoserine phosphatases [17]. It is known that phosphoserine phosphatase (PSP) is essential for serine and glycine metabolism [18], [19] but no PSP is annotated for *Synechococcus*. The alternative pathways for serine and glycine synthesis in cyanobacteria were proposed [20], [21], however, we tested if one or more of the PGMs annotated in *Synechococcus* can be identified as PSP. Cluster analysis of PGM-like proteins shows that each PGM belongs to different protein family (Fig. 6) but, interestingly, PSPs in *Hydrogenobacter thermophilus* are in the same cluster as PGM3 (Fig. 6). Moreover, we have detected conservative regions between that PSPs and PGM3. It is therefore likely that we can identify PGM3 as PSP, however, we cannot entirely exclude that this enzymes functions as PGM as well. Finally, PGM3 is up-regulated in low CO_2_ (Fig. 3) which lead to speculation on contribution of photorespiration for serine biosynthesis as suggested [20].

As for the PGM1 and PGM4, due to almost the same expression in low CO_2_ (Fig. 2), we do not have sufficient data to clearly distinguish between these two down-regulated enzymes. We however showed that stronger down-regulation gives higher robustness to the system, as indicated in Fig. 3. There might also be other reasons why there are three, or even four, PGMs in *Synechococcus*. For instance, it is known that the majority of proteins have more than one function [22].

The simultaneous parameter estimation for two PGMs for more steady states is very difficult and nearly impossible for three PGMs. One can therefore assume a rather small likelihood for the right combination of substrate-product affinities for every PGM to occur during evolution. Moreover, our analysis shows that the system in not very sensitive to varying separately the affinity of beta or gamma PGM although the best fit fulfills the expectation regarding the affinities for the product/substrate for two isoenzymes (Tab. 1). Therefore, an occurrence of several PGMs could be explained, from the evolutionary point of view, as the simplest means to achieve the robust response of the regulated system. As we have shown, the number of isoenzymes has a big impact on the system robustness (Fig. 5). Indirect support for such speculation is our analysis for triple PGMs scenario where k_M_ values, both for beta and gamma PGMs, were varied. The results demonstrate even higher robustness of the system (Fig. 5, green line) than the case of fixed beta PGM and varied gamma PGM (Fig. 5, blue line). Furthermore, the mean quality of match with experimental data improved by 5.6% in comparison to dual reverse PGMs scenario. Taken together, these results might imply that in the case of essential metabolic crossroads, the occurrence of more than two isoenzymes catalyzing the same reaction, especially in non-controlled environment, work as a buffer keeping the homeostasis of the system.

Despite the fact that our analysis cannot provide a clear cut answer why there are more PGMs, the presented approach contributes to an understanding of enzymatic regulation and provides a ration approach to identify the roles of particular, even non-homologous, isoenzymes.

## Supporting Information

Model S1 The model of primary carbon metabolism in cyanobacterium *Synechococcus elongatus* PCC 7942 is provided and encoded in SBML L2V4 and set up for triple PGMs scenario, based on the best fits of alpha, beta and gamma PGM (Fig. 4).
(XML)
